# Structural integrity of the insula and emotional facial recognition performance following stroke

**DOI:** 10.1093/braincomms/fcad144

**Published:** 2023-04-28

**Authors:** Kai Klepzig, Martin Domin, Julia Wendt, Bettina von Sarnowski, Alexander Lischke, Alfons O Hamm, Martin Lotze

**Affiliations:** Functional Imaging Unit, Institute of Diagnostic Radiology and Neuroradiology, University Medicine Greifswald, Walther-Rathenau-Str.46, 17475 Greifswald, Germany; Functional Imaging Unit, Institute of Diagnostic Radiology and Neuroradiology, University Medicine Greifswald, Walther-Rathenau-Str.46, 17475 Greifswald, Germany; Department of Psychology, University of Potsdam, Karl-Liebknecht-Straße 24-25, 14476 Potsdam, Germany; Department of Neurology, University Medicine Greifswald, Sauerbruchstrasse, 17475 Greifswald, Germany; Department of Psychology, Medical School Hamburg, Am Kaiserkai 1, 20457 Hamburg, Germany; Institute of Clinical Psychology and Psychotherapy, Medical School Hamburg, Am Kaiserkai 1, 20457 Hamburg, Germany; Biological and Clinical Psychology, University of Greifswald, Franz-Mehring-Straße 47, 17475 Greifswald, Germany; Functional Imaging Unit, Institute of Diagnostic Radiology and Neuroradiology, University Medicine Greifswald, Walther-Rathenau-Str.46, 17475 Greifswald, Germany

**Keywords:** facial emotional recognition, insula, brain lesion mapping, white-matter integrity, stroke

## Abstract

The role of the human insula in facial emotion recognition is controversially discussed, especially in relation to lesion-location-dependent impairment following stroke. In addition, structural connectivity quantification of important white-matter tracts that link the insula to impairments in facial emotion recognition has not been investigated. In a case–control study, we investigated a group of 29 stroke patients in the chronic stage and 14 healthy age- and gender-matched controls. Lesion location of stroke patients was analysed with voxel-based lesion-symptom mapping. In addition, structural white-matter integrity for tracts between insula regions and their primarily known interconnected brain structures was quantified by tractography-based fractional anisotropy. Our behavioural analyses showed that stroke patients were impaired in the recognition of fearful, angry and happy but not disgusted expressions. Voxel-based lesion mapping revealed that especially lesions centred around the left anterior insula were associated with impaired recognition of emotional facial expressions. The structural integrity of insular white-matter connectivity was decreased for the left hemisphere and impaired recognition accuracy for angry and fearful expressions was associated with specific left-sided insular tracts. Taken together, these findings suggest that a multimodal investigation of structural alterations has the potential to deepen our understanding of emotion recognition impairments after stroke.

## Teaser

Klepzig *et al*. reported impairment in recognition of the facial expression of fear and anger in patients with left insula damage caused by stroke. Decreased white-matter integrity of tracts reaching from the left insula in these patients was associated with a reduced recognition of fear and anger expressions.

## Graphical Abstract

**Graphical abstract:**
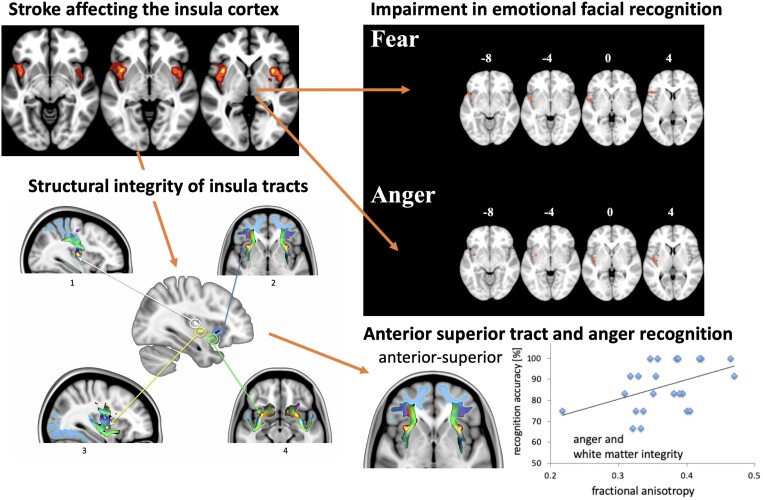

## Introduction

Patients surviving severe brain damage following stroke show social impairments that are unrelated to an actual degree of physical impairment.^1^ Social integration is an important factor for improved functional outcome^2^ and therefore the consideration of social-cognitive disturbances is of particular importance when treating deficits following stroke.

Given that facial expressions communicate the emotional states and intentions of individuals,^3^ the correct recognition of emotional expressions is crucial for successful social interaction. Such abilities can be easily assessed in experimental settings, typically using pictures of prototypical facial expressions of emotions.^4^ Studies using these tasks described impairments in facial emotion recognition in stroke patients at both chronic and subacute stages.^5,6^ Moreover, impaired emotion recognition accuracy was associated with social withdrawal,^7^ relationship dissatisfaction^8^ and problematic changes in behaviour such as losing one’s temper or impulsivity in stroke cohorts.^9^

The insular cortex seems to be crucially involved in facial emotion recognition,^10^ while disgust has been traditionally linked to this structure.^11^ Lesion studies suggest that the left insula is particularly involved in the recognition of disgust.^12,13^ However, findings derived from single case reports on lesions covering the left insula are not always consistent, either failing to show significant impairments in recognizing disgust from facial expressions^14^ or failing to show any impairment of emotion recognition in faces in general even when the insula is bilaterally damaged.^15^ A mostly preserved recognition of emotional expressions, including disgust, has also been reported in a patient with a right-sided insula lesion.^16^ Similarly, a patient with a right-sided insula lesion showed rather subtle deficits in emotion recognition, which emerged only during the processing of expression with low emotional intensity.^17^ Another patient with a right insula lesion showed impairment only during the processing of negative expressions, indicating once again selective rather than global impairments in emotion recognition.^18^ A fundamental drawback of single case reports refers to the extent of brain damage that mostly comprises further sites next to the lesion of interest, which hampers drawing adequate conclusions. For example, impaired recognition accuracy, especially for expressions of disgust, was found in a patient with bilateral insula lesions caused by Herpes simplex; however, the lesions also affected the other parts of the brain extensively.^19^

While these case studies provided valuable insight into insula-dependent impairments in emotion recognition, studies with larger patient samples are warranted to better understand the association between insula lesions and emotion recognition after a stroke. In addition, studies that assess emotion recognition performance and relate the function explicitly to insular lesions following stroke are scarce. Actually, in recent approaches, lesions were not restricted to the insular cortex, while results on lesion laterality and recognition impairments were inconsistent.^20,21^

Due to these inconsistent findings, the nature of emotion recognition impairments in patients with insular lesions remains elusive. So far, available data suggest that recognition impairments are more pronounced in patients with left insular lesions, particularly for negative emotions that are more difficult to recognize than, e.g. happy faces.^22^ In addition, all these studies only investigated lesions but did not investigate white-matter changes that often impair function following stroke in a crucial way.^23^ Given that white-matter tracts connect the insula with other brain areas that are implicated in emotion processing, it is conceivable that white-matter alterations in these tracts are associated with impairments in emotion recognition.^17^ When considering the high interaction of the insula for cognitive, emotional, somatosensory/pain and body integration processing, it becomes evident that the structural integrity of interconnecting tracts is of high interest for emotional processing of the insula.

We performed a prospective case–control study to investigate facial emotion recognition in chronic stroke patients with brain lesions covering or sparing the insula. Whereas previous studies were limited by methods that provided a coarse spatial resolution of insular lesions,^24^ we applied voxel-based lesion-symptom mapping (VLSM) analyses to describe in more detail the association between specific lesion sites and emotion recognition impairments.^25^ In addition, we used diffusion-weighted imaging (DWI) to investigate whether white-matter alterations were also associated with emotion recognition impairments. We used fractional anisotropy (FA) to quantify white-matter integrity and compared a possible decrease in FA with healthy controls (HCs). For those tracts that showed relevant impaired structural connectivity for the stroke patients, we investigated possible associations of tract FA with their facial emotion recognition performance. This correlation analysis was again restricted to those emotions with recognition impairment.

## Materials and methods

### Participants

We performed a case–control study in patients and HCs matched for age. Selection criteria of stroke patients comprised lesions covering parts of the insula, or lesions sparing the insula. Finally, we examined 53 patients suffering from ischaemic or haemorrhagic stroke, recruited from the stroke unit of the University Medicine Greifswald from March 2015 to March 2019. Patients were included when being adult (age >18 years), in the chronic stage after stroke (at least 5 months) with diagnosed initial impairment and stroke diagnosis based on among others the initial CT or MRI scan of the brain performed in the first week after stroke. The exclusion criteria were age >90 years, the presence of cognitive deficits reported by attending physicians or relatives, schizophrenia and a history of neurodegenerative disorders, epilepsy, brain traumas or tumours. Supplementary Fig. 1 provides a flow chart depicting exclusions and drop outs of participants. Finally, data of 29 patients (lesions: 15 for left-hemispheric, 11 for right-hemispheric, 1 for bilateral, 1 for pons, 1 for cerebellum) were used for group comparisons and lesion mapping analyses (see Table 1). For the DWI analyses, data from 23 stroke patients were available. Data of 14 age-matched HCs with no history of diagnosed neurological or psychiatric disorders recruited through community advertisement were used for comparison. All considered patients and HCs reported normal or corrected-to-normal vision.

**Table 1:** Participant characteristics

	Healthy controls	Stroke patients	Statistics
No. of participants	14	29	
Age, years	62.3 (15.6)	64.2 (13.5)	*t* = −0.408, *P* = 0.685
Gender (F:M)	8:6	9:20	*P* = 0.182
Handedness (L:R)	0:14	2:27	*P* = 1.000
Months since stroke		34.1 (35.7)	* *
Lesion volume, cm^3^		21.7 (27.6)	
School years	10.3 (1.3)	10.0 (1.2)	*t* = 0.789, *P* = 0.435
MWT-B	29.1 (3.6)	27.7 (5.1)	*t* = 0.931, *P* = 0.357
Simple reaction, ms	360 (68)	361 (74)	*t* = −0.017, *P* = 0.986
AAT	55.6 (4.4)	54.1 (4.0)	*t* = 1.108, *P* = 0.274
CVLT	52.2 (12.4)	46.8 (13.1)	*t* = 1.299, *P* = 0.201
TMT B/A	2.5 (0.8)	2.5 (1.0)	*t* = −0.201, *P* = 0.842
STROOP, s	86.2 (21.8)	101.1 (28.0)	*t* = −1.746, *P* = 0.088
Benton	7.1 (1.5)	6.0 (1.6)	*t* = 2.002, *P* = 0.052
FAB	94.3 (7.3)	93.1 (8.1)	*t* = 0.464, *P* = 0.645
BDI	9.0 (7.9)	7.2 (6.2)	*t* = 0.805, *P* = 0.426

Mean values are presented with standard deviation in brackets. F, female; M, male; L, left; R, right; MWT-B, number of correctly recognized words; AAT, score of the verbal comprehension task; CVLT, number of all correctly remembered nouns of the California Verbal Learning Task; TMT B/A, time required for the TMT-B/time required for the TMT-A; STROOP, time required for the STROOP interference task; Benton, number of correct drawings; FAB, percentage of correct trials in the facial identity discrimination task; BDI, sum score of the Beck Depression Inventory II.

The study was conducted according to the standards as defined in the Declaration of Helsinki and had been approved by the Ethics Committee of the University Medicine Greifswald (BB29/09b). All participants signed written informed consent forms and were financially compensated.

### Procedure

The study presented here was part of a comprehensive examination that spanned 3 days within 1 week. On the first day, the participants were invited to the Department of Psychology of the University of Greifswald, where they were briefed about the experimental procedures. After providing written informed consent, the participants completed various questionnaires and neuropsychological tests. On the second day, the MRI investigation was performed at the MRI facility of the Department of Radiology of the University Medicine Greifswald. On the third day, the emotion recognition experiment was conducted at the Department of Psychology of the University of Greifswald. Participants were seated in a comfortable chair with arm rests in a dimly lit and sound-attenuated room. Stimulus presentation was realized with a computer screen 1.5 m in front of them. After the experimental procedure, the participants were debriefed.

### Demographical, neuropsychological and psychopathological measures

The National Institute of Health Stroke Scale score,^26^ assessed at admission to the stroke unit (available for 25 patients), was obtained from medical records. Information on handedness, education (school years) and clinical characteristics including diagnoses and age of lesion (in months) were also obtained. As cognitive deficits are relatively common after a stroke,^27^ a variety of neuropsychological tests were administered in order to examine various facets of brain functions: verbal intelligence with the German multiple-choice word test MWT-B,^28^ alertness with the simple reaction task of the NeuroCogFX software,^29^ verbal comprehension with the Aachen Aphasia Test,^30^ verbal memory with the German version of the California Verbal Learning Test,^31^ executive functioning with the Trail Making Tests A (numbers) and B (numbers and letters) of the Consortium to Establish a Registry for Alzheimer’s Disease-Plus,^32^ susceptibility to interference with the German version of the Stroop Colour-Word Interference Test,^33^ visuospatial memory with the Benton Visual Retention Test,^34^ facial blindness with a computerized adaption of the facial identity discrimination task of the Florida Affect Battery^35^ using pictures showing neutral facial expressions taken from the FACES database.^36^ Depression, which is quite prevalent among stroke patients,^37^ was assessed with the Beck Depression Inventory (BDI) II.^38^ One patient failed to complete the entire questionnaire and was therefore not considered for a comparison of BDI scores.

### Emotion recognition task

For the emotion recognition task, pictures showing faces of 12 individuals (6 women, 6 men) with fearful, angry, happy, disgusted and neutral expressions were selected from a validated face database.^36^ Following an established procedure,^39^ the selected faces were converted into grey scales, normalized in luminance and equalized in size using Photoshop CS4 (Adobe Systems Inc., San Jose, CA, USA) and Matlab 7.7 (MathWorks Inc., Natick, MA, USA). In addition, faces were enclosed in an elliptic mask to minimize the influence of expression-irrelevant features on task performance. The faces were shown in a pseudo-random order to the participants who had to provide an emotion rating and intensity rating for each face. Each trial consisted of a baseline phase when a fixation cross was shown for a duration of 3 s, a viewing phase when a face was shown for a duration 6 s, and a rating phase when an emotion and an intensity scale were shown for a self-determined duration. The emotion rating was done on a scale comprising five emotion labels (German words for angry, happy, fearful, disgusted and neutral), and the intensity rating was done on a scale ranging from 1 (low) to 9 (high). The emotion labels and the rating scale appeared consecutively at the bottom of the screen while the face was visible all the time. Participants were instructed to rate as promptly as possible using the dominant hand. Twelve pictures were used for each expression (6 female faces) resulting in 60 stimuli. Presentation of stimuli and recording of ratings and response times were realized using Presentation software (Neurobehavioral Systems, Berkeley, CA, USA; version 16.1).

### Magnetic resonance imaging

A 3T MRI scanner (Siemens Verio, Erlangen, Germany) with a 32-channel (DWI) and a 12-channel (T_1_- and T_2_*-weighted scans as the basis for lesion mapping) head coil was used to record MRI data. T_1_-weighted imaging for lesion mapping was carried out using a sagittal 3D MPRAGE with 176 slices, a spatial resolution of 0.98 × 0.98 × 1 mm^3^. The field of view was 250 × 250 mm^2^, corresponding to an acquisition matrix of 256 × 256. Repetition time was 1690 ms, echo time was 2.52 ms and total acquisition time was 230 s. For T_2_*-weighted imaging, we used a Flair sequence with 45 axial slices of 3 mm thickness with 0.6 mm gap, field of view: 220 mm corresponding to an acquisition matrix of 320 × 240; repetition time was 10 000 ms; echo time 107 ms; total acquisition time 162 s. Lesions were manually drawn by an experienced neuroscientist/neurologist (M.L.) using MRIcron^40^; on structural T_1_-weighted images, adding the Flair images if necessary for lesion localization, and confirmed by medical reports. For DWI, we used the standard multidirectional diffusion–weighted Siemens sequences. A protocol with 64 diffusion-weighting gradient directions with a *b*-value of 1000 s/mm^2^ and one reference image with no diffusion weighting (*b* = 0 s/mm^2^) was selected (72 axial slices; 1.8 × 1.8 × 2 mm^3^ voxel size; matrix size 128 × 128; TR 10 900 ms; TE 107 ms; total measurement time 770 s).

### DWI preprocessing

**Figure 1:**
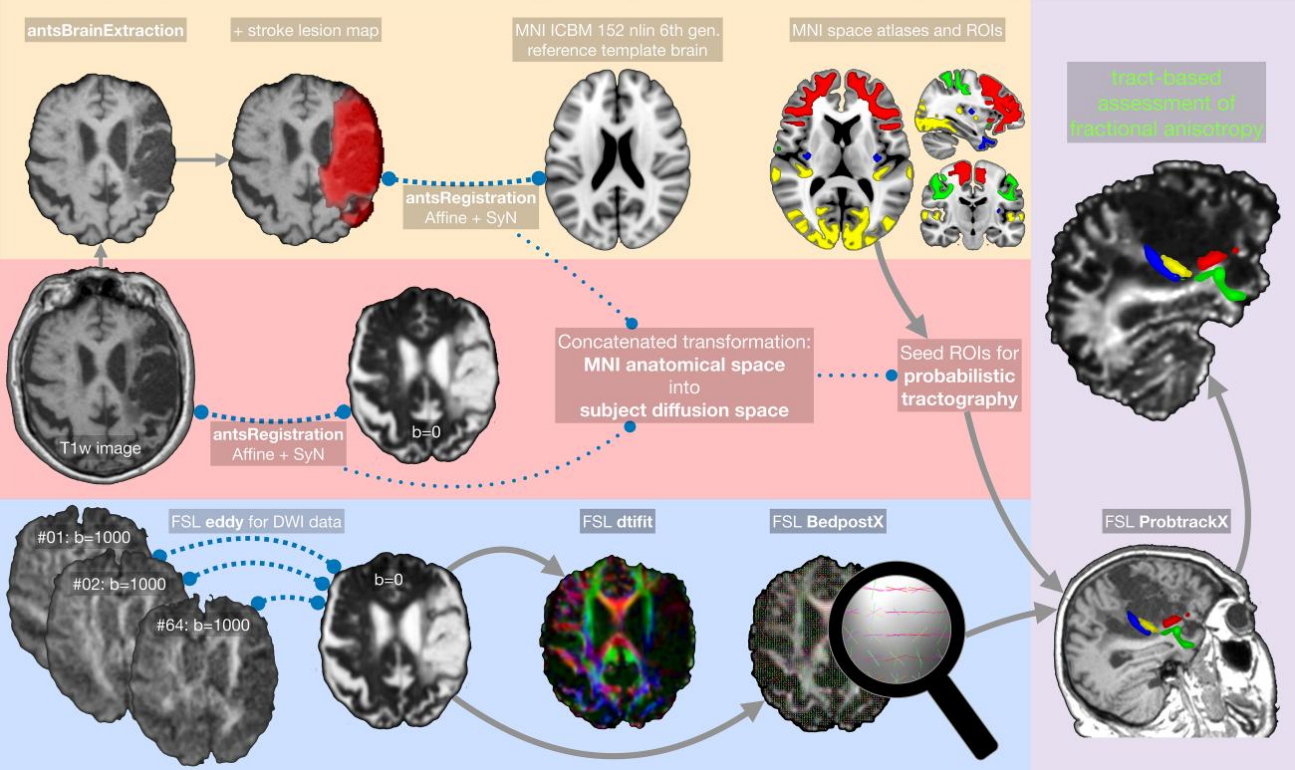
**Demonstration of the method for evaluating the white-matter integrity from insula tracts.** Data processing was performed using FSL (v6.0.4, Analysis Group, FMRIB, Oxford, UK) and the ANTs (v2.3.5.dev212-g44225).^41^ Upper row: T_1_-weighted (T1w) images were brain-extracted and (non-)linearly registered to the Montreal Neurologic Imaging template space (MNI152 NLIN 6th gen.), including the patients’ lesion masks to improve registrations. Middle row: T1w images were (non-)linearly registered to the DWI b0 image. A concatenated transformation (MNI → T1w → DWI) for transformation of regions of interest (ROIs) or atlases from MNI space into subject space was calculated. Lower row: Eddy current-related distortions and subject movement in the DWIs were corrected and outlier slices were replaced. The diffusion tensor and diffusion-related indices, e.g. FA, were calculated (FSL dtifit), as well as distributions on diffusion parameters at each voxel (FSL BedpostX). Right column: The transformed ROIs were used to compute a connectivity distribution by using probabilistic tractography. The resulting tractograms were used for a tract-based assessment of FA.

Diffusion-weighted data and anatomical T_1_-weighted data were processed as described in Fig. 1. Afterwards a quality check took place utilizing FMRIB Software Library (FSL)’s eddy QC tools^42^ and all QC-related parameters, such as subject movement, were well within required limits.

### Regions-of-interest for probabilistic tractography

**Figure 2:**
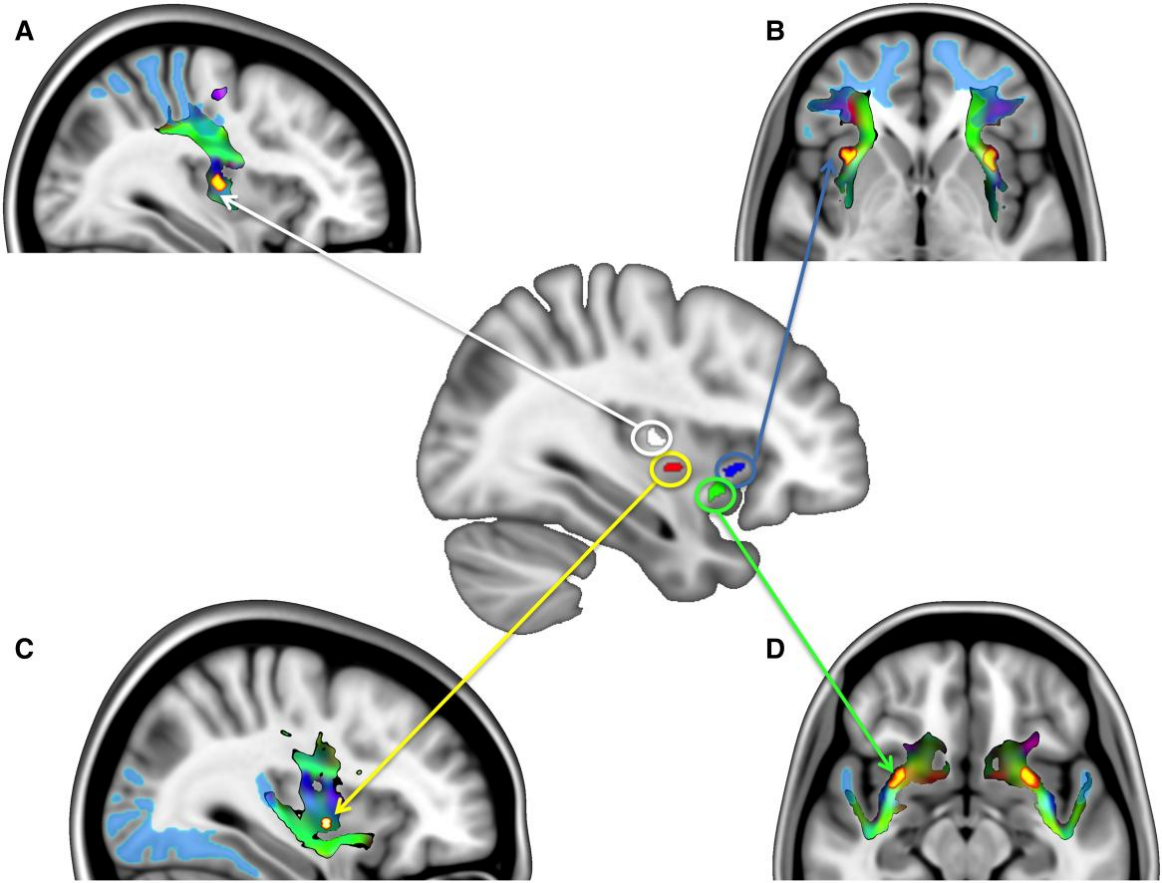
**Location of the four insula seeds, tracks and grey matter regions used for the FA analysis.** Location of the four insula seeds in the centre of the figure shown on one hemisphere and the tractography from seeds to the grey matter ROIs projected into grey matter (coloured in light blue) (surrounding pictures **A–D**). Tractograms are depicted as colour-coded frequency maps (DTI eigenvector direction coding: *x*-red, *y*-green, *z*-blue; seed ROI in the four surrounding slices indicated by red-yellow. The following seeds have been tested: (**A**) the superior posterior insula seed (white ROI) tracking to the primary somatosensory cortex and superior parietal lobe (top left), (**B**) the superior anterior insula seed (blue ROI) tracking into the lateral PFC (top right), (**C**) the inferior posterior insula seed (red ROI) tracking to the inferior temporal and the occipital cortex (visual discriminative tract; bottom left), and (**D**) the inferior anterior insula seed (green ROI) tracking into the temporal pole (bottom right).

Ghaziri *et al*.^43^ used parcellation of both insula cortices in 19 subregions to define cortical projections from these seeds by using high-angular resolution diffusion imaging tractography on 46 young healthy adults. That study revealed a wide array of connections between the insula and the frontal, temporal, parietal and occipital lobes. We condensed their findings to the four main seeds (anterior dorsal and ventral and posterior dorsal and ventral), projecting to the corresponding cortical brain areas using existing atlas-based masks [automated anatomic labelling toolbox: lateral prefrontal cortex (PFC), temporal pole; Anatomy Toolbox: SPL, occipital and inferior temporal lobe]. Figure 2 illustrates the four different regions of interest (ROIs) per hemisphere.

### Data analyses

#### Statistical analysis of participant characteristics

Group differences regarding demographic characteristics, neuropsychological deficits and depressive mood were investigated using independent sample *t*-tests. Distributions of gender and handedness were compared with Fisher’s exact tests.

#### Statistical analysis of emotion recognition performance

Recognition accuracy (in %), mean response time for correct recognition and mean intensity ratings were calculated for each expression and investigated for group differences using repeated measures (rms) ANOVAs with *Group* (stroke patients, HC) as between-subjects factor and *Expression* (fear, anger, disgust, happiness, neutral) as within-subjects factor. In case of significant main effects, *post hoc* comparisons with Holm–Bonferroni corrections were computed. Greenhouse–Geisser corrections were applied, when the assumption of sphericity was violated. All analyses comprising participant characteristics and recognition performance were conducted with IBM SPSS Statistics 22 (Armonk, NY, USA) using an Alpha of 0.05. Bee swarm plots were created using R and the bee swarm package.^44,45^ Since rmANOVAs showed that response time and intensity ratings were unaffected in the stroke cohort, further examinations (i.e. VLSM, FA) were solely focused on recognition accuracy.

#### Statistical analysis of lesion mapping (VLSM)

For the VLSM analysis (including all stroke patients), the Advanced Normalization Tools (ANTs, v2.2.0)^41^ were used to register and spatially normalize the T_1_-weighted images into Montreal Neurologic Institute (MNI) space (MNI ICBM152 6th generation; see Fig. 1). The individual lesion mask images were transformed into MNI space by applying the same transformation using the GenericLabel-Interpolator. Using the Non-Parametric Mapping Toolbox (delivered with MRIcron) for each facial expression, voxel-wise Brunner–Munzel rank tests were performed to compare recognition accuracy between stroke patients having a lesion in that voxel and stroke patients having no lesion in that voxel. Computations were realized for whole-brain and ROI analyses (ROI: bilateral insular cortex). The insular cortex map was created using the Neuromorphometrics atlas (Neuromorphometrics, Inc.; http://neuromorphometrics.com/). Only voxels lesioned in at least 15% of the participants (*n* = 4) were included. To correct for multiple comparisons, threshold *Z*-values were adjusted using the false discovery rate correction as previously suggested.^25^ Alpha was set at 0.05.

#### Statistical analysis of white-matter integrity (FA quantification)

Prior to analysis, FA of the four considered tracts was averaged for each hemisphere. Hemisphere-specific group differences in FA were investigated using an rmANOVA with *Tracts* (four seeds) and *Side* (left hemisphere, right hemisphere) as within-subjects factors and *Group* (stroke patients, HC) as between-subjects factor. Pearson correlations were applied only for stroke patients restricted on (i) the hemisphere that showed relevant FA differences between groups and (ii) group differences in facial recognition performance. Again, a multiple comparison correction was performed with the Holm–Bonferroni method. Analyses of FA were conducted with IBM SPSS Statistics 22.

## Results

### Participant characteristics

**Figure 3:**
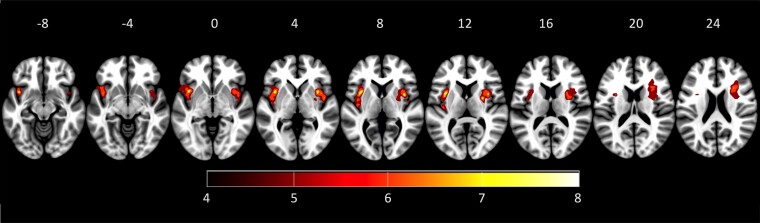
**Lesion mapping of stroke patients included.** Sum lesion map of all stroke patients included (*n* = 29; colour coding below). Numbers above axial slices indicate *z*-positions of the MNI coordinates. The right hemisphere is shown on the right (neurological view).

Patients had a median Nation Institute of Health Stroke Scale score of 4 at admission and performed similarly to HCs on neuropsychological tests (see Table 1). Lesion summation maps indicated the predominant location of lesions in the left or right insula (see Fig. 3).

### Recognition accuracy

**Figure 4:**
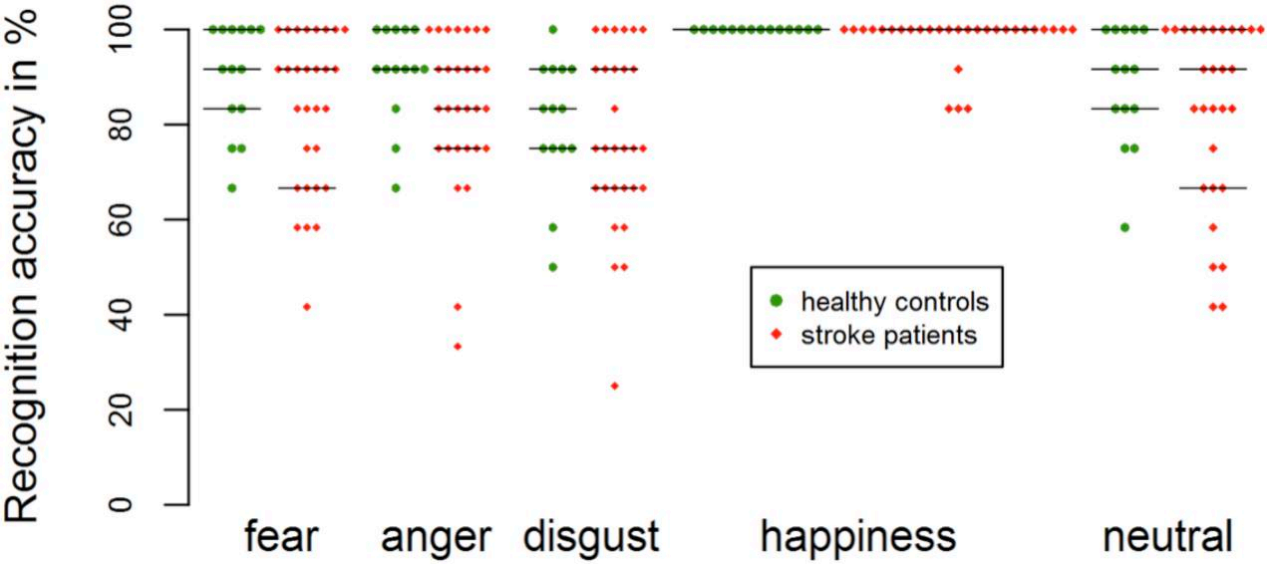
**Behavioural data for facial recognition accuracy.** Swarm plots showing individual recognition accuracy of HCs (green circles) and stroke patients (red squares). Horizontal lines represent median values and first and third quartiles.

Figure 4 provides a bee swarm plot showing individual recognition accuracy respectively for each expression. The rmANOVA investigating differences in emotion recognition revealed a significant effect of *Expression* [*F*(4,164) = 12.273, *P* < 0.001, $\eta_{p}^{2}$ = 0.230] in the absence of an *Expression* × *Group* interaction [*F*(4,164) = 0.346, *P* = 0.805, $\eta_{p}^{2}$ = 0.008], indicating expression-specific differences in emotion recognition across both groups. Happy expressions were generally better recognized than any other expression (all *P-*values <0.01), whereas angry expressions were slightly better recognized than disgust expressions (*P* = 0.084). There was also a marginally significant effect for *Group* [*F*(1,41) = 3.043, *P* = 0.089, $\eta_{p}^{2}$ = 0.069], indicating that stroke patients were generally worse in emotion recognition than HCs. Using one-sided independent sample *t*-tests for exploratory follow-up analyses revealed that stroke patients were especially impaired in the recognition of happy expressions (*P* = 0.025) and slightly impaired in the recognition of fearful (*P* = 0.085) and angry expression (*P* = 0.051) compared with HCs. Table 2 provides an overview of participants’ emotion recognition accuracy. Examinations of response times for correct recognitions showed a significant effect of *Expression* [*F*(4,164) = 4.370, *P* = 0.002, $\eta_{p}^{2}$ = 0.096] while no interaction between *Expression* and *Group* could be found [*F*(4,164) = 1.173, *P* = 0.325, $\eta_{p}^{2}$ = 0.028], which indicates differences between expressions regardless of group. Fearful expressions were recognized more slowly than neutral (*P* = 0.020) and happy expressions (*P* = 0.036). The patients’ response times did not significantly differ from those of HCs [*F*(1,41) = 1.717, *P* = 0.197, $\eta_{p}^{2}$ = 0.040].

**Table 2:** Comparisons of recognition accuracy

	Healthy controls	Stroke patients	
	Mean (SD)	Mean (SD)	Statistics
Fear	89.9 (11.4)	83.0 (16.4)	*t* = 1.398, *P = 0*.085
Anger	91.1 (10.1)	83.0 (16.4)	*t* = 1.676, *P = 0*.051
Disgust	80.4 (13.7)	77.0 (18.7)	*t* = 0.595, *P = 0*.278
Happiness	100 (0.0)	98.0 (5.3)	*t* = 2.045, *P* = 0.025
Neutral	88.1 (12.5)	82.8 (19.4)	*t* = 0.936, *P* = 0.178

Recognition performance (accuracy in %) of HCs and patients for emotional and neutral expressions. Mean values are presented with standard deviation in parentheses. Single comparisons using independent sample *t*-tests are listed (one-tailed *P*-values are reported, since poorer recognition is expected in patients).

### Intensity ratings

Analyses of intensity ratings revealed a significant effect of *Expression* [*F*(4,164) = 51.293, *P* < 0.001, $\eta_{p}^{2}$ = 0.556] in the absence of an interaction between *Expression* and *Group* [*F*(4,164) = 2.258, *P* = 0.113, $\eta_{p}^{2}$ = 0.052] showing that intensity ratings differed between expressions for both groups. Neutral expressions were rated as less intense compared with all emotion expressions (all *P-*values <0.01). No significant effect of *Group* could be found which indicates that intensity ratings of stroke patients were comparable with those of HCs [*F*(1,41) = 0.305, *P* = 0.583, $\eta_{p}^{2}$ = 0.007].

### Voxel-based lesion-symptom mapping

**Figure 5:**
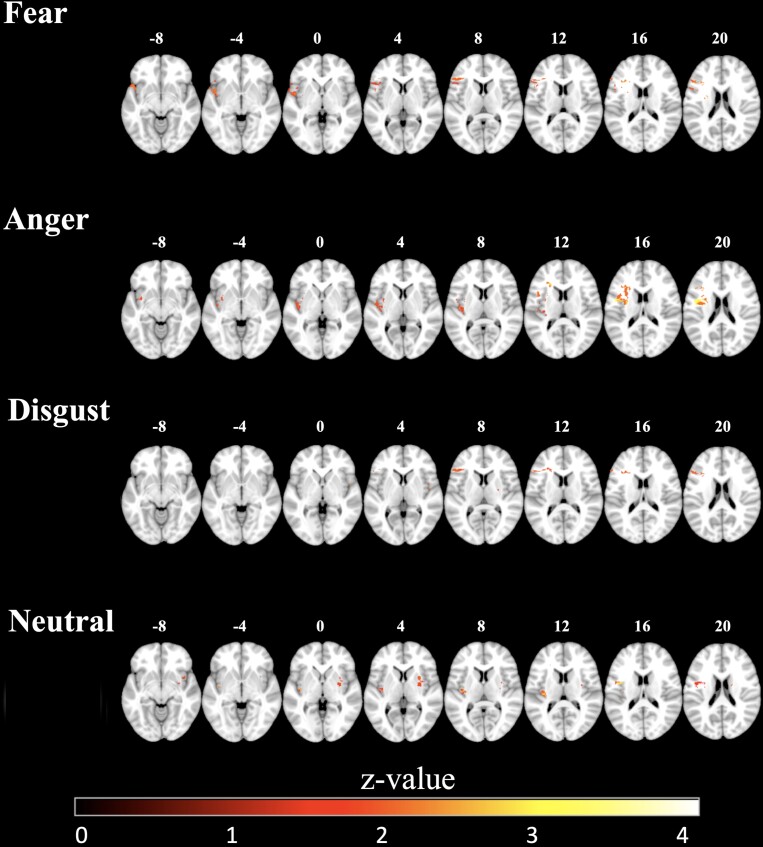
**Demonstration of the results of voxel-based lesion-symptom mapping.** Demonstration of the results of VLSM (*P* < 0.05 uncorrected for display purpose) in a group of 29 stroke patients corresponding to the respective emotional expression. Left column: examples for emotional faces presented. Location of transverse slices is provided as *Z*-values in MNI coordinates on the top. Colour coding for statistical values is indicated at the bottom of the figure. For expressions of happiness, no critical lesion sites could be found and therefore, it is not depicted here. For presentation of results corrected for multiple comparisons within the insula volume, see Table 3.

An explorative analysis with VLSM (whole brain, not corrected for multiple comparisons, see Fig. 5) demonstrated that impairments in emotion recognition were predominantly associated with brain lesions centred around the left insula: impaired recognition of fear was associated with lesions of the left lateral insula cortex; impaired recognition of anger was associated with lesions of the left superior anterior and inferior posterior insula; impaired recognition of disgust was associated with lesions of the left ventrolateral PFC, more anterior to the anterior insula; impaired recognition of neutral expressions was associated with lesions of the left posterior insula. No critical sites were found for the recognition of happiness. After correcting for multiple comparisons in a ROI analysis (bilateral insular cortex), impairments in the recognition of fear, anger and neutral expressions remained significantly associated with voxel clusters in the left insular cortex (see Table 3). Impairments in disgust recognition, on the contrary, were no longer significantly associated with voxel clusters in the insular cortex.

**Table 3:** Lesion sites within grey matter associated with impaired recognition accuracy (ROI analysis)

					MNI coordinates		
Expression	Hemisphere	Number of voxels	Peak voxel	*Z*-value	*x*	*y*	*z*
Fear	Left	58	Ant IC	2.118	−43	5	−5
Anger	Left	101	Ant IC	2.466	−33	−1	14
Neutral	Left	341	Post IC	2.658	−40	−9	9

Coordinates are reported for the peak region and refer to MNI space. Only voxels with significant *Z-*values corrected for multiple comparisons are considered. *Z*-value, maximum *Z-*value found in the peak region; ant, anterior; post, posterior; IC, insular cortex.

### Diffusion-weighted imaging

**Figure 6:**
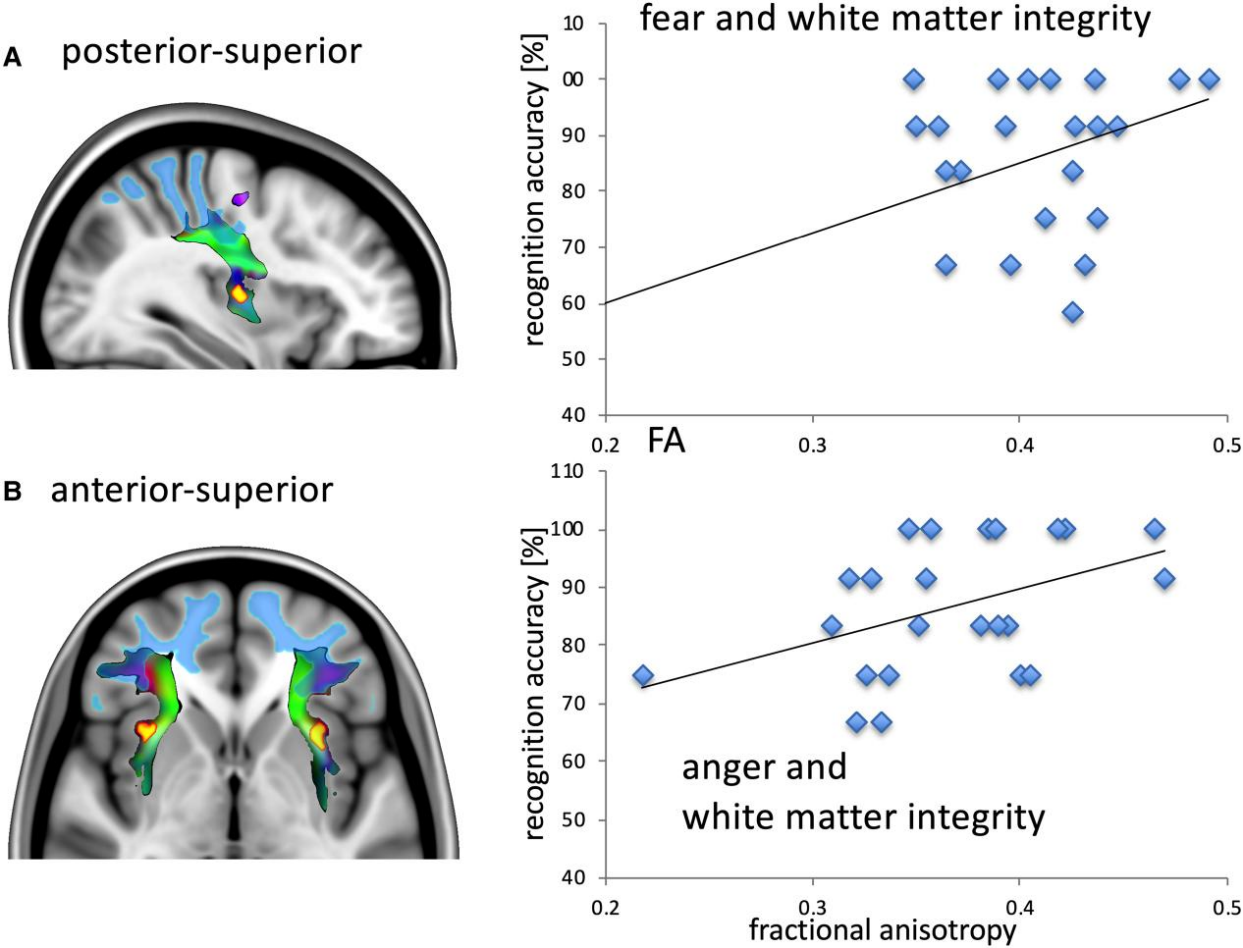
**Results from the analysis of white-matter quantification.** Left column illustration of the tracts (seed yellow; tract blue-red-green) and their cortical projections (illustrated in light blue). Right column shows plots of the FA data for the left hemisphere, respectively (linear regression). (**A**) Association of the FA of the left parietal tract from the posterior-superior insula seed with fear recognition accuracy. Regression line provided. (**B**) Association of the FA of the left prefrontal tract from the anterior-superior insula seed with anger recognition accuracy. Regression line provided.

The rmANOVA investigating differences in FA revealed a significant effect of *Group* [*F*(1,35) = 6.203, *P* = 0.018, $\eta_{p}^{2}$ = 0.151], a significant effect of *Tracts* [*F*(3,105) = 73.502, *P* ≤ 0.001, $\eta_{p}^{2}$ = 0.677], and a significant *Side*–*Tracts* interaction [*F*(3,105) = 11.599, *P* ≤ 0.001, $\eta_{p}^{2}$ = 0.249] which suggests that stroke patients generally have reduced white-matter integrity compared with HC and that side differences vary according to the considered tract for both patients and controls. No significant effect of *Side* could be found [*F*(1,35) = 1.461, *P* = 0.235, $\eta_{p}^{2}$ = 0.040]. Exploratory follow-up analyses using independent sample *t*-tests revealed primarily lower FA in stroke patients for the left hemisphere [*T*(35) = 2.443; *P* = 0.04, corrected for two comparisons]. When investigating group differences in FA of each of the four tracts of the left hemisphere, we found decreased FA for the tract connecting the left inferior posterior insula seed to the occipitotemporal cortex [*T*(35) = 3.13; *P* = 0.016, corrected for four comparisons; Fig. 6A].

Association analyses were only performed for those emotions relevantly impaired (fear, anger; *n* = 2) but showed no ceiling effect (happy). Tracts were restricted to those of the left hemisphere, since FA decrease was restricted to the left in our patient group (*n* = 4). We expected a positive association of FA in the patient group and facial recognition accuracy. The association of FA between the left posterior dorsal insula seed and parietal lobe with fear recognition stood significance for multiple comparisons between the number of tracts tested [*r*(23) = 0.538; *P* = 0.032, corrected for eight comparisons]. However, the association of FA in left anterior dorsal seed projecting to the dorsolateral PFC (DLPFC) with fear and anger recognition was also high [fear: *r*(23) = 0.425; *P*_uncorr_ = 0.022; anger: *r*(23) = 0.449; *P*_uncorr_ = 0.016].

## Discussion

In the present work, stroke patients at chronic stage were examined using a facial emotion recognition paradigm while recognition accuracy was assessed. Analyses comprised (i) a group comparison between stroke patients and carefully matched HCs, (ii) a VLSM to investigate the specificity of lesioned brain regions for facial recognition impairments and (iii) the quantification of damage in white-matter integrity of four distinct insular seeds per hemisphere. All participants were comprehensively screened to assure comparability between patients and HCs.

Assessment of lesions revealed that they frequently covered parts of the left or right insula (see Fig. 1), confirming our criteria for patient recruitment. In tracts originating from the insula, DWI revealed lower FA in stroke patients than in HCs. More specifically, FA for the inferior posterior insula seed interconnecting to visual processing areas in the occipital and inferior temporal lobe was decreased. Hence, compared with HCs, the examined patient cohort showed relevant lesions of the insula and its projections to other brain regions.

### Behavioural parameters/facial emotion recognition

Stroke patients showed slight impairments in emotion recognition in general. These impairments appeared to be particularly pronounced during the processing of fearful and angry expressions resulting in trend significance. Fearful and angry expressions are generally difficult to discriminate,^46^ and impaired recognition for these emotions is typically found in stroke patients.^5,47^ The recognition of happy expressions was also impaired, although it was generally better than the recognition of all other expressions. Similar findings have been reported in a previous study.^5^ Stroke patients were not impaired during the processing of disgusted expressions, which is in line with previous findings^6,48^ though also impaired capabilities have been reported.^49^ Stroke patients showed normal response times, which suggests that correct recognitions did not simply result from prolonged evaluation or decision making processes and, hence, that impairments in our stroke cohort were rather mild. A further support for the latter impression is that our patients showed intensity ratings comparable with those of HC indicating preserved sensitivity to facial expressions. The finding of only subtle impairments might be explained by the fact that the examined patient cohort suffered from rather less severe strokes. In fact, in a previous approach on patients with mild strokes also rather, small impairments were found,^6^ while lesion size was found to be negatively correlated with recognition accuracy.^20^

### Associations between emotion recognition impairments and lesion location

A VLSM whole-brain analysis revealed that recognition of neutral, fearful, angry and disgusted expressions in patients was associated with lesion location in the left insula. The associations involving neutral, fearful and angry expressions remained significant after correcting for multiple comparisons in a VLSM ROI analysis (Table 3). Recognition impairments for happy expressions were not associated with lesion mapping. This may be due to the fact that the recognition of happy expressions was generally high, implying that the restricted range of variance preclude the detection of associations (ceiling effects). These findings are in line with previous studies reporting emotion recognition impairments in patients with lesions of the left insula.^14,50,51^

### Associations of white-matter damage with recognition impairment

Mirroring the VLSM findings in the left insula, the DWI analyses revealed associations between impaired recognition of angry and fearful expressions with white-matter alterations in tracts originating from the left insula. The highest association was observed for the tracts originating from left dorsal posterior insula. Impairments in the recognition of fearful expressions were associated with white-matter alterations in tracts that connected the insula to the superior parietal lobe. The superior parietal lobe is critical for visuospatial processing^52^ and face processing,^53^ possibly implying that alterations in these regions lead to impairments in emotion recognition. The association for fearful and angry facial expressions with FA to the left DLPFC showed also medium effect sizes. An interaction of the DLPFC with insula function in emotional control and recognition of emotionally evocative stimuli had been described before.^54^

### General discussion

In the present study, we demonstrated that considering both lesioned brain regions and white-matter alterations in networks of brain regions is a fruitful approach when studying emotion recognition impairments in stroke patients. First, our results corroborate Boucher *et al*.^50^ who found intact disgust recognition in patients with unilateral insular surgery; however, they contradict previous findings of impaired disgust recognition in patients with left insular lesions.^12,13^ Hence, the current work do not point to a specific role of the insula and rather supports a more comprehensive involvement in the processing of emotional expressions as previously suggested.^55^ Likewise, the revealed associations of left insular cortex lesions with impaired recognition are in line with results of a meta-analysis on functional imaging data and face processing.^10^ Second, impairments in recognition of neutral expressions were associated with lesions in the left posterior insula. Therefore, our finding of varying insular regions being critical for the recognition of emotional and non-emotional faces is in line with a recent work using intracranial recordings which showed that posterior parts were generally associated with processing faces, while the anterior insula responded solely to behaviourally relevant face stimuli (i.e. faces showing emotional expressions).^55^

Generally, the insula has a central function in the perception and evaluation of body signals, which is highly critical for the activation of subjective experiences of emotional states but also for the encoding of behaviourally relevant information such as facial expressions.^56^ The anterior and posterior regions of the insula differ not only in terms of their cytoarchitecture and functions, but also regarding their afferent and efferent connections. While the posterior part of the insular cortex is connected to the thalamus, the parietal cortex and the superior temporal gyrus, the anterior insula maintains close connections to the limbic system, particularly to the amygdala, the anterior cingular and PFC and the basal ganglia.^57^ Regarding functions, the posterior insula is responsible for the representation of various visceral functions and physiological body processes^56^ and integrates these information with those transmitted by an emotionally relevant stimulus (e.g. an observed face) prior to a subjective assessment.^55^ In contrast, the anterior insula is more involved in the evaluation of these body processes in terms of personal relevance regardless of valence, which then forms the basis for various emotional and motivational states.

Therefore, according to our data, it appears that lesions especially in the left insular cortex and associated tracts reaching to somatosensory areas in the parietal cortex cannot that easily be compensated and result in an impaired processing of those emotional expressions that are generally difficult to recognize due to a distorted integration of body signals and emotional responses.

The finding of especially prominent associations between lesions (i.e. insula and associated tracts) and impaired recognition of fearful expressions appears plausible as the anterior insula was shown to be in particular responsive towards such expressions,^55^ which might reflect their outstanding relevance.^58^ In contrast, posterior lesions of the insula rather seem to spare the processing of emotionally relevant stimuli which underlines previous conceptualizations of distinct insular subregions with varying functions. Lesions of the right insula might not affect emotion recognition as also previously shown^16^; however, associated tracts to the PFC appear critical.

### Limitations

Our study comprised a series of examinations that were performed on several days at different research facilities. This decreased the number of patients that could be included (we had already selected patients for a time period of >5 years). For some of our statistical procedures, such as VLSM, the number of patients included is relatively low, allowing only moderate-to-strong effects to survive. This was the reason for a ROI analysis of our VLSM approach, which is a recommended strategy when dealing with smaller samples.^59^ Particularly for the white-matter tract associations with the investigated four tracts, the power was definitely too low to allow for multiple comparison correction. However, this is the first insula lesion study, investigating emotional recognition impairment in association with white-matter integrity, applying multiple comparison correction. In addition, given the exhaustive nature of the study design, we cannot rule out that especially highly motivated and well-recovered stroke patients were motivated to participate in our study, indicating a self-selection bias.^6^ Accordingly, our stroke patients showed much smaller lesion sizes (mean = 21.7 cm^3^) than patients of a recent study.^21^ We investigated a less disturbed sample of stroke patients, and this may explain why we found more selective impairments in emotion recognition than other studies.^5^ Another reason for the selective impairments in emotion recognition may be related to the use of VLSM. Using VLSM, only those brain areas can be examined that are lesioned in a sufficient number of participants. As lesions were not equally distributed among our stroke patients, we cannot rule out that the brain regions for which we found no association are, in fact, related to facial emotion recognition. However, as we used the insula cortex as a target region and included enough patients with lesions covering this region, the VLSM was an adequate strategy to investigate associations between emotion recognition impairments and structural alterations.

## Conclusion

To our knowledge, this is the first study to examine facial emotion recognition performance including analyses of lesion-symptom mapping and white-matter integrity in stroke patients with left or right insula lesions. Our results show that selective impairments in facial emotion recognition are present even in well-recovered patients with less severe strokes. Moreover, we show that these deficits are especially related to lesions of the left insular cortex and associated tracts. Therefore, a major part of the processing of facial emotional recognition seems to be lateralized to the left hemisphere. Our findings corroborate previous findings of clinical case reports and imaging studies on healthy participants, implying a general relevance of the insula in emotion recognition beyond disgust processing. However, our findings suggest only moderate impairments in emotional processing of facial expressions in patients with insula lesions. In the light of well-documented negative consequences for the life of patients after brain lesions of various aetiologies, we suggest not only that more weight should be placed on emotion recognition impairments in the treatment of strokes in general, but, especially, that patients with left lesions of the insular cortex should be seen as a high-risk group who are particularly prone to emotional disturbances.

## Supplementary Material

fcad144_Supplementary_Data

## Data Availability

Lesion maps, tables of recognition performance and FA averages for the selected tracts can be downloaded from https://github.com/martinlotze/insula_stroke_facial_recognition.

## Abbreviations

BDI =: Becks depression inventory
DLPFC =: dorsolateral prefrontal cortex
DWI =: diffusion-weighted imaging
FA =: fractional anisotropy
FSL =: FMRIB Software Library
HC =: healthy controls
MNI =: Montreal Neurologic Institute
ROI =: regions of interest
VLSM =: voxel-based lesion-symptom mapping
