# Supplementary figures and images for: Structural integrity of the insula and emotional facial recognition performance following stroke
Source: Brain Commun. 2023 Apr 28;5(3):fcad144. doi: 10.1093/braincomms/fcad144 (PMC10244053; doi:10.1093/braincomms/fcad144)

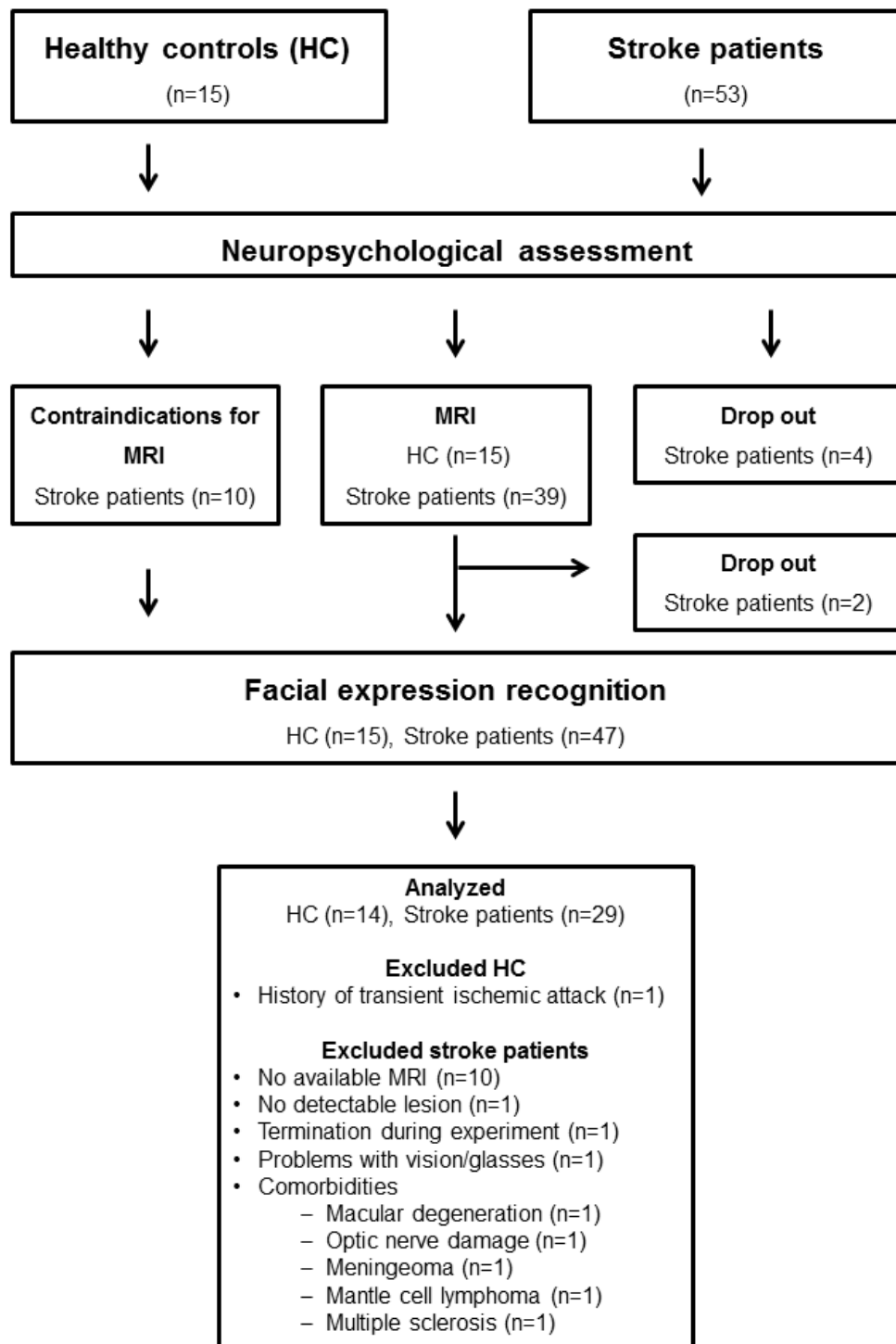

Supplementary Figure 1: Flow chart for the study

Supplement: fcad144_Supplementary_Data [file fcad144_supplementary_data.pdf]
